# Risk factors and machine learning prediction models for bronchopulmonary dysplasia severity in the Chinese population

**DOI:** 10.1007/s12519-022-00635-0

**Published:** 2022-11-10

**Authors:** Wen He, Lan Zhang, Rui Feng, Wei-Han Fang, Yun Cao, Si-Qi Sun, Peng Shi, Jian-Guo Zhou, Liang-Feng Tang, Xiao-Bo Zhang, Yuan-Yuan Qi

**Affiliations:** 1 Department of Respirology, Children’s Hospital, Fudan University, 399 Wanyuan Road, Shanghai, 201102 China; 2 Department of Neonatology, Children’s Hospital, Fudan University, Shanghai, China; 3 Shanghai Key Laboratory of Intelligent Information Processing, School of Management and Statistics, Fudan University, Shanghai, China; 4 Shanghai Pinghe Bilingual School, Shanghai, China; 5 Department of Data Management and Statistics, Children’s Hospital of Fudan University, Shanghai, China; 6 Department of Urology, Children’s Hospital, Fudan University, Shanghai, China

**Keywords:** Bronchopulmonary dysplasia, Machine learning, Prediction model, Preterm

## Abstract

**Background:**

Bronchopulmonary dysplasia (BPD) is a common chronic lung disease in extremely preterm neonates. The outcome and clinical burden vary dramatically according to severity. Although some prediction tools for BPD exist, they seldom pay attention to disease severity and are based on populations in developed countries. This study aimed to develop machine learning prediction models for BPD severity based on selected clinical factors in a Chinese population.

**Methods:**

In this retrospective, single-center study, we included patients with a gestational age < 32 weeks who were diagnosed with BPD in our neonatal intensive care unit from 2016 to 2020. We collected their clinical information during the maternal, birth and early postnatal periods. Risk factors were selected through univariable and ordinal logistic regression analyses. Prediction models based on logistic regression (LR), gradient boosting decision tree, XGBoost (XGB) and random forest (RF) models were implemented and assessed by the area under the receiver operating characteristic curve (AUC).

**Results:**

We ultimately included 471 patients (279 mild, 147 moderate, and 45 severe cases). On ordinal logistic regression, gestational diabetes mellitus, initial fraction of inspiration O_2_ value, invasive ventilation, acidosis, hypochloremia, C-reactive protein level, patent ductus arteriosus and Gram-negative respiratory culture were independent risk factors for BPD severity. All the XGB, LR and RF models (AUC = 0.85, 0.86 and 0.84, respectively) all had good performance.

**Conclusions:**

We found risk factors for BPD severity in our population and developed machine learning models based on them. The models have good performance and can be used to aid in predicting BPD severity in the Chinese population.

**Supplementary Information:**

The online version contains supplementary material available at 10.1007/s12519-022-00635-0.

## Introduction

Bronchopulmonary dysplasia (BPD) is a chronic respiratory disease originating in the neonatal period and a major complication of prematurity. With the development of neonatology and the wide use of pulmonary surfactant (PS), the pathology of BPD has changed greatly. However, BPD still accounts for a significant extent of preterm morbidity and mortality. Additionally, BPD can cause numerous long-term sequelae in many aspects [1, 2]. The prognosis of BPD depends on its severity. Patients with mild severity perform as well as their non-BPD peers in terms of lung function, while patients with severe disease always have more severe airway obstructions, and patients with moderate severity are in between these two patient groups [3]. Moreover, poor short-term as well as long-term neurodevelopmental outcomes occur in patients with BPD, especially in those with moderate and severe BPD [4, 5].

Despite more than 50 years of research, there is a lack of effective treatment options for BPD [6, 7]. According to the National Institute of Child Health and Human Development (NICHD) diagnosis criteria, the determination of BPD severity cannot be decided until postmenstrual age (PMA) 36 weeks. Thus, the early prediction of BPD severity is worth attention, as it can predict the clinical burden and guide treatment and follow-up strategies.

Several researchers have developed prediction tools, with clinical, imaging, genomic, or even more complicated biomarkers as factors, using traditional statistics or machine learning models to identify infants at high risk of BPD [8–11]. Most of the existing models focus on the early prediction of BPD; few pay attention to BPD severity, although the three levels of severity show different consequences and prognoses. In addition, most of the tools were established in developed countries based on European–American populations, which differ in race and clinical treatment guidelines from Chinese populations. Therefore, current tools may not perform well in China.

With the advantage of being able to detect a very small causal relation among data, machine learning models are promising prediction tools and have been used in many clinical applications over the years [12]. A variety of machine learning algorithms have been applied to develop decision models used to help clinical diagnosis and treatment. In the present study, we aimed to identify the clinical risk factors for BPD severity and develop an effective early prediction model for BPD severity based on machine learning techniques in the Chinese population.

## Methods

### Patients

**Fig. 1:**
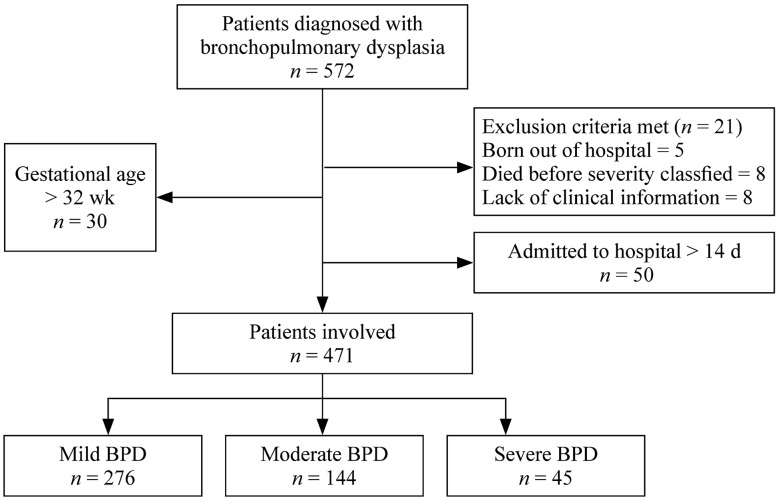
Flow chart of the study showing the process of the model development, from the patients’ involvement and the filtration of risk factors to the development and assessment of prediction models based on the machine learning algorithm. *BPD* bronchopulmonary dysplasia

This retrospective, single-center study was conducted at a level IV neonatal intensive care unit (NICU) in a tertiary children hospital in Shanghai, China. All premature infants with a gestational age (GA) < 32 weeks who were admitted to the Children’s Hospital of Fudan University from Jan 01 2016 to Dec 31 2020 and diagnosed with BPD during hospitalization were eligible. After excluding patients with incomplete clinical data and patients who were admitted to the hospital after 14 days of life, 471 patients remained for the final analysis. We also involved all 1032 preterm infants with GA < 32 weeks without BPD in the same period, and their detailed data are described in the supplementary file (Supplementary Table 1). This study was approved by the Ethics Committee of the Children’s Hospital of Fudan University (Ethics approval number: 2021–122). Informed consents were obtained from their parents of the participants. The study flowchart is shown in Fig. 1.

### Potential predictor variables

As the etiology of BPD is complex, all clinical factors during antenatal, perinatal, and early postnatal life were considered. In addition to well-known risk factors, such as GA, birth weight and sex, we also evaluated clinical factors shown to be related to BPD recently, such as C-reactive protein (CRP) [13].

Data on maternal and birth characteristics were collected from the medical history taken at admission. Respiratory support [initial mode as continuous positive airway pressure (CPAP), initial fraction of inspiration O_2_ (FiO_2_), invasive ventilation], laboratory examination results (routine blood test, blood-gas test, culture results), complications [patent ductus arteriosus (PDA), necrotizing enterocolitis (NEC)] and treatment (caffeine, corticosteroid treatment, antibiotics exposure without culture, prolonged antibiotics exposure) were obtained from the electronic medical database. Airway specimens included tracheal aspirates and broncho-alveolar lavage fluid. Routine blood and blood gas tests were performed as clinically needed. All the above clinical information was collected within the first 14 days of life. We adopted the maximum CRP level and white blood cell count (WBC), as well as the minimum pH value, sodium concentration, and chloride concentration as variables for statistical analysis.

Gestational diabetes mellitus (GDM) was defined as any degree of glucose intolerance during pregnancy, regardless of the presence of diabetes before pregnancy [14]. Maternal hypertensive disorder was defined as a mean blood pressure (BP) ≥ 140 mmHg and/or diastolic BP ≥ 90 mmHg more than once during pregnancy [15]. Early-onset neonatal sepsis (EOS) and late-onset sepsis (LOS) were defined as bacteremia occurring within 72 hours and after 72 hours, respectively [16]. Small-for-gestational-age was diagnosed by a birthweight below the 10th percentile for the GA at birth, in accordance with Fenton chart [17]. PDA was diagnosed by color-flow Doppler echocardiography. NEC was diagnosed according to the modified Bell’s staging criteria [18]. Positive airway specimen culture was defined as the presence of bacteria other than “normal airway flora” on culturing and was classified by Gram staining. Antibiotic exposure without culture was defined within the first week of life, while prolonged antibiotics were defined as antibiotic exposure without culture started in the first week and lasted for more than five days.

### Definition of study outcomes

We referred to the 2001 NICHD criteria for model development [19]. The primary outcome was BPD severity categorized as mild, moderate, or severe based on respiratory support at a postmenstrual age of 36 weeks. Mild BPD was defined as breathing room air, moderate BPD was defined as FiO_2_ < 0.30, and severe BPD was defined as FiO_2_ ≥ 0.30 or positive pressure ventilation at a postmenstrual age of 36 weeks.

### Data analysis

SPSS version 20.0 (IBM Corporation, Armonk, New York, USA) was used for data analysis. Numerical variables are expressed as the mean ± standard deviation (SD) or as the median with percentiles 25–75 (P25–P75). Normally distributed variables were analyzed using the analysis of variance, while non-normally distributed variables were analyzed using the Kruskal-Wallis *H* test. For variables with significant *P* values on the Kruskal-Wallis *H* test, we applied the non-parametric Mann-Whitney *U* test for further pairwise comparisons. Categorical data were compared using the chi-square test. We entered factors with a *P* value < 0.10 into the regression test. All continuous covariates were centered on the mean and scaled to the standard deviation. Ordinal logistic regression was used for univariate and multivariate analyses. Variance inflation factor (VIF) coefficients were reviewed to avoid collinearity. All risk factors with statistical significance in multivariate analysis (*P* value < 0.05) were used to develop the prediction model. Moreover, all the selected risk factors were compared between preterm infants without BPD and all BPD infants to prove evidence.

### Model development

Machine learning modeling was implemented using R 4.4.1 statistical software (R Foundation for Statistical Computing, Vienna, Austria). Patients were randomly split into a training set (80%) and test set (20%). Fivefold cross-validation was used to improve the models. Four types of algorithms were used to determine the optimal strategy and model: gradient boosting decision tree (GBDT), logistic regression (LR), XGBoost (XGB) and random forest (RF). Receiver operating characteristic (ROC) curves were plotted, and the area under the ROC curve (AUC) was used to evaluate the diagnostic value of each model.

## Results

### Demographics and clinical burden of the patients

A total of 471 infants with BPD were enrolled. The demographics and clinical burden of the patients are shown in Table 1. Among all the patients, 279 were mild (59.2%), 147 moderate (31.2%), and 45 severe (9.6%). The mean GA was 28.0 ± 1.6 (27.0, 29.0) weeks, and the mean birth weight (BW) was 1136 ± 248 (960, 1280) g. BW and GA were significantly lower for severe BPD than for mild and moderate BPD. Severe BPD patients had the longest hospital stay as well as oxygen therapy, while mild BPD patients had the shortest and moderate were in between. All the patients had high medical costs, but severe patients had highest cost, which is about 1.5 times higher than mild patients.

**Table 1:** Demographic and clinical burden in the patients with mild, moderate and severe bronchopulmonary dysplasia (BPD)

Variables	Mild (*n* = 279)	Moderate (*n* = 147)	Severe (*n* = 45)	*P* value
GA, wk, mean (SD)	28.0 (1.3)^†^	28.3 (1.8)^‡^	27.5 (2.1)	0.002^§^
BW, g, mean (SD)	1146 (216)^†^	1193 (322)^‡^	1098 (346)	0.001^§^
Male, *n* (%)	174 (62.4)	85 (57.8)	27 (60.0)	0.656
SGA (< P10), *n* (%)	6 (2.2)	6 (4.1)	3 (6.7)	0.210
Length of hospital stay, d, mean (SD)	66 (16)^*,†^	76 (25)^‡^	89 (40)	< 0.001^§^
Length of oxygen support, d, mean (SD)	45.0 (14.2)	63.2 (19.8)	89.8 (44.8)	< 0.001^§^
Corrected age at discharge, wk, mean (SD)	37.6 (1.9)^*,†^	40.0 (2.6)	40.5 (4.3)	< 0.001^§^
Total cost, thousand yuan, mean (SD)	165 (56)^*,†^	200 (80)^‡^	250 (114)	< 0.001^§^

*GA* gestational age, *BW* birth weight, *SGA* small-for-gestational-age, *SD* standard deviation, *P10* 10th percentile

^*^P < 0.05, comparison between mild and moderate

^†^*P* < 0.05, comparison between mild and severe

^‡^*P* < 0.05, comparison between moderate and severe

^§^Comparison between the groups with significance

The risk factors during the antenatal and perinatal periods are shown in Table 2. On univariable analysis, patients with severe BPD were more likely to suffer from maternal GDM and less likely to have antenatal corticosteroid treatment than the others. No significant differences were observed among the severity groups in the presence of maternal hypertensive disorder, maternal age, or delivery mode. After birth, the five-minute Apgar score and intubation rates were significantly different among the severity groups. Infants with severe BPD were more likely to undergo intubation in the delivery room (DR) than infants with mild or moderate BPD.

**Table 2:** Antenatal and birth characteristics in the patients with mild, moderate and severe bronchopulmonary dysplasia (BPD)

Variables	Mild (*n* = 279)	Moderate (*n* = 147)	Severe (*n* = 45)	*P* value
Antenatal corticosteroid treatment, *n* (%)	180 (64.5)^†^	79 (53.7)^‡^	23 (51.1)	0.045^§^
GDM, *n* (%)	34 (12.2)^†^	28 (19.0)^‡^	11 (24.4)	0.040^§^
Maternal hypertensive disorder, *n* (%)	46 (16.5)	21 (14.3)	11 (24.4)	0.277
Maternal age, y, mean (SD)	31.6 (4.6)	31.1 (4.5)	31.5 (5.0)	0.600
Delivery by cesarean section, *n* (%)	139 (49.8)	82 (55.8)	16 (35.6)	0.058
1-min Apgar score, median (P25–P75)	7 (5–8)	7 (5–8)	6 (4–8)	0.230
5-min Apgar score, median (P25–P75)	8 (8–9)^†^	8 (7–9)	8 (6–8)	0.027^§^
Intubation in the delivery room, *n* (%)	83 (27.1)^*,†^	55 (30.2)^‡^	28 (44.4)	0.024^§^

*GDM* gestational diabetes mellitus, *SD* standard deviation, *P25–P75* percentiles 25–75

^*^P < 0.05, comparison between mild and moderate

^†^*P* < 0.05, comparison between mild and severe

^‡^*P* < 0.05, comparison between moderate and severe; §comparison between the groups with significance

The clinical characteristics in early postnatal life are shown in Table 3. Patients with severe BPD needed the highest FiO_2_ initially, while the mild group needed the lowest FiO_2_ among the three groups. Patients with severe BPD had the highest WBC and CRP levels as well as the lowest pH value among the three groups. Patients with severe BPD were also more likely to suffer from PDA than those with mild or moderate BPD. Early treatment was different among the different groups. Patients with moderate BPD were more likely to have postnatal corticosteroid treatment than patients with mild BPD. In addition, patients with severe BPD were more likely to be exposed to prolonged antibiotics without a positive blood culture. Regarding infections, patients with severe BPD were more likely to have LOS and positive respiratory specimen culture than patients with mild or moderate BPD.

**Table 3:** Early postnatal clinical characteristics of the patients of mild, moderate and severe bronchopulmonary dysplasia (BPD)

Variables	Mild (*n* = 279)	Moderate (*n* = 147)	Severe (*n* = 45)	*P* value
Laboratory test				
WBC-max, × 10^3^/μL, mean (SD)	25.6 ± 15.3^†^	25.1 ± 10.5^‡^	31.4 ± 11.7	0.001^§^
CRP-max, g/L, mean (SD)	20.4 ± 27.6^*,†^	30.5 ± 36.1^‡^	56.9 ± 54.1	< 0.001^§^
PH-min, mean (SD)	7.23 ± 0.08^*,†^	7.20 ± 0.08^‡^	7.16 ± 0.10	< 0.001^§^
Na-min, mmol/L, mean (SD)	130.3 ± 3.7^†^	129.9 ± 3.9^‡^	128.1 ± 4.1	0.001^§^
Cl-min, mmol/L, mean **(**SD)	99.0 ± 4.9^*,†^	97.0 ± 5.0^‡^	92.7 ± 14.6	< 0.001^§^
Complications				
PDA, *n* (%)	183 (65.6)^†^	106 (72.1)	38 (84.4)	0.027^§^
NEC, *n* (%)	22 (7.9)	18 (12.2)	8 (17.8)	0.077
Neonatal sepsis-				
Early onset, *n* (%)	22 (7.9)	11 (7.5)	3 (6.7)	0.956
Late onset, *n* (%)	77 (27.6)^*,†^	44 (29.9)^‡^	23 (51.1)	0.006^§^
Respiratory specimen culture				
Gram + , *n* (%)	81 (29)^*,†^	61 (41.5)	24 (53.3)	0.001^§^
Gram − , *n* (%)	101 (36.2)^*,†^	71 (48.3)^**‡**^	35 (77.8)	< 0.001^§^
Early treatment				
Caffeine, *n* (%)	272 (97.5)	138 (93.9)	43 (95.6)	0.176
Postnatal corticosteroid treatment, *n* (%)	28 (10.0)^*^	33 (22.4)	9 (20.0)	0.002^§^
Antibiotics exposure without culture, *n* (%)	246 (88.2)	131 (89.1)	35 (77.8)	0.109
Prolonged antibiotics exposure, *n* (%)	128 (45.9)^*,^^**†**^	83 (56.5)	28 (62.2)	0.031^§^
Respiratory support				
Initial FiO_2_, %, mean (SD)	29.6 ± 12.8^*,^^†^	34.5 ± 14.4^‡^	37.0 ± 18.0	< 0.001^§^
Initial CPAP, *n* (%)	75 (26.9)^†^	35 (23.8)^‡^	4 (8.9)	0.032^§^
Invasive ventilation, *n* (%)	153 (54.8)^*^	83 (56.5)^‡^	37 (82.2)	0.002^§^

*WBC* white blood cell counts, *max* maximum, *min* minimum, *CRP* C-reactive protein, *PH* pH value, *Na* sodium concentration, *Cl* chloride concentration, *PDA* patent ductus arteriosus, *NEC* necrotizing enterocolitis, *CPAP* continuous positive airway pressure, *FiO*_*2*_ fraction of inspiration O_2_, *Gram* + Gram-positive bacteria, *Gram − * Gram-negative bacteria, *SD* standard deviation

^*^P < 0.05, comparison between mild and moderate

^†^*P* < 0.05, comparison between mild and severe

^‡^*P* < 0.05 between moderate and severe

^§^Comparison between the groups with significance

### Multivariable analysis

The results of the ordinal logistic regression analysis are shown in Table 4. Severity was entered as the outcome, and patients with mild, moderate, and severe disease were designated as 1, 2, and 3, respectively. Maternal diabetes mellitus, initial FiO_2_ value, invasive ventilation, acidosis, hypochloremia, C-reactive protein level, patent ductus arteriosus and Gram-negative respiratory culture were independent risk factors for BPD severity. The calculated VIFs varied between 1.019 and 1.154 (no VIF > 10), which indicated the absence of collinearity between the variables. Moreover, all the factors were significantly different between the BPD group and the non-BPD group (Table 5). We also supplemented the selected risk factors for a preliminary model based on the 2018 NICHD definition [20]. The odds ratios are shown in Supplementary Table 2; ROC of the machine learning model is shown in Supplementary Fig. 1, and detailed indexes are shown in Supplementary Table 3.

**Table 4:** Results of the ordinal logistic regression model for bronchopulmonary dysplasia (BPD) severity levels

Characteristics	Crude odds ratio^a^ 95% CI	*P*^a^ value	Adjusted odds ratio^b^ 95% CI	*P*^b^ value
GDM	1.700 (0.920, 3.140)	< 0.001	2.272 (1.080, 4.780)	0.031
Initial FiO_2_	1.030 (1.013, 1.047)	< 0.001	1.032 (1.012, 1.052)	< 0.001
Invasive ventilation	1.875 (1.142, 3.080)	0.013	2.593 (1.400, 4.802)	0.002
PH-min	0.001 (0.000, 0.012)	< 0.001	0.005 (0.001, 0.189)	0.005
Cl-min	0.899 (0.856, 0.944)	< 0.001	0.904 (0.851, 0.961)	0.001
CRP-max	1.027 (1.018, 1.035)	< 0.001	1.026 (1.016, 1.036)	< 0.001
Gram – in respiratory culture	2.878 (1.755, 4.720)	< 0.001	2.311 (1.273, 4.197)	0.006
PDA	3.150 (1.733, 5.727)	< 0.001	6.328 (2.937, 13.631)	< 0.001

*GDM* gestational diabetes mellitus, *FiO*_*2*_ fraction of inspiration O_2_, *PH* pH value, *min*, minimum, *Cl-* blood chloride concentrations, *CRP* C-reactive protein, *max* maximum, *Gram* – Gram-negative bacteria, *PDA* patent ductus arteriosus, *95% CI* 95% confidence interval

^a^Crude odd ratio and *P* values are of ordinal logistic regression

^b^Adjusted odds ratios and *P* value for gestational age, gender and birth weight

**Table 5:** Risk factors between the very preterm infants with and without bronchopulmonary dysplasia (BPD)

Variables	Non-BPD (*n* = 1032)	BPD (*n* = 471)	*P* value
GDM, *n* (%)	112 (10.9)	73 (15.5)	0.014^*^
CRP-max, g/L, mean (SD)	11.6 ± 15.5	26.8 ± 34.6	< 0.001^*^
PH-min, mmol/L, mean (SD)	7.30 ± 0.07	7.21 ± 0.08	< 0.001^*^
Cl-min, mmol/L, mean (SD)	104.5 ± 5.0	97.8 ± 6.7	< 0.001^*^
Gram– in respiratory culture, *n* (%)	35 (3.4)	207 (43.9)	< 0.001*
Initial FiO_2_, %, mean (SD)	24.77 ± 7.7	31.72 ± 14.3	< 0.001^*^
Initial CPAP, *n* (%)	341 (33)	114 (24.2)	0.001^*^
Invasive ventilation, *n* (%)	358 (34.7)	273 (58.0)	< 0.001^*^
PDA, *n* (%)	430 (41.7)	327 (69.4)	< 0.001^*^

*GDM* gestational diabetes mellitus, *FiO*_*2*_ fraction of inspiration O_2_, *PH* pH value; min, minimum, *Cl-* blood chloride concentrations, *CRP* C-reactive protein, *max*, maximum, *Gram – *Gram-negative bacteria, *PDA* patent ductus arteriosus, *CPAP* continuous positive airway pressure, *SD* standard deviation

^*^Comparison between the groups with significance

### Model performance

**Fig. 2:**
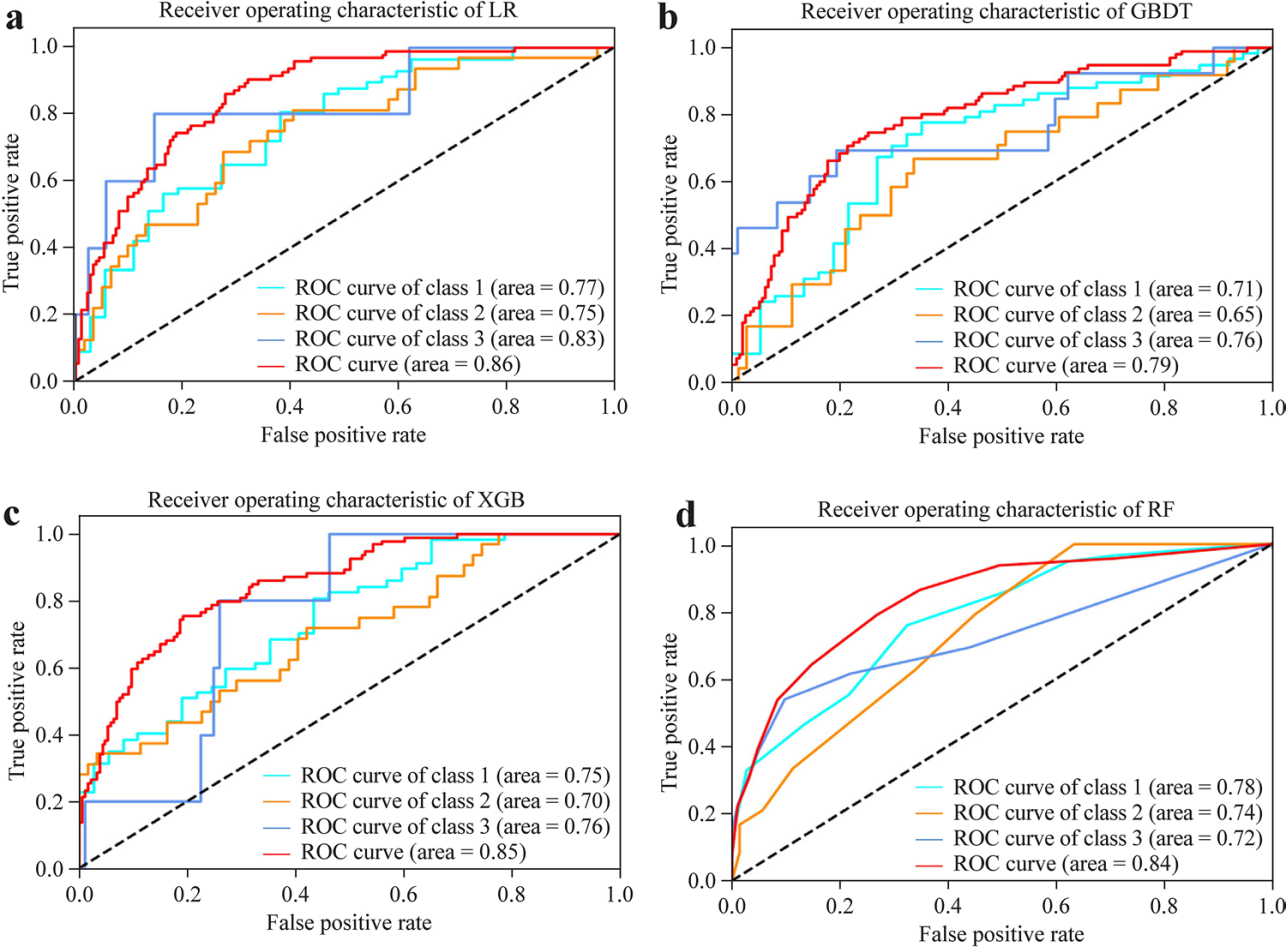
Performance of different machine learning models showing the comparisons for different learning algorithms on test sets. **a–d** The ROC curves of the LR, GBDT, XGB and RF models. Class 1 represents mild BPD; class 2 represents moderate BPD, and class 3 represents severe BPD. *LR* logistic regression, *RF* random forest, *GBDT* gradient boost decision tree, *ROC* receiver operating characteristic, *AUC* area under the receiver operating characteristic curve, *BPD* bronchopulmonary dysplasia

All eight clinical factors with significant differences on multivarible analysis as well as key factors (GA and BW) were entered into the machine learning models. ROC curves were drawn for the test set (Fig. 2). The AUCs of the GBDT, XGB, RF, and LR models were 0.79 ± 0.045 (95% CI 0.70–0.88), 0.85 ± 0.043 (95% CI 0.77–0.94), 0.84 ± 0.038 (95% CI 0.77–0.91) and 0.86 ± 0.036 (95% CI 0.79–0.93), respectively. Thus, the LR, XGB and RF models all had good performance in this database. The detailed indexes for the four models are presented in the supplemental material (Supplementary Table 4).

## Discussion

The differences among various severities of BPD have been intensively investigated in recent years. Patients with severe BPD have worse prognoses and require more intensive care and follow-up. The prediction of severe BPD is of great importance for patients with BPD. Currently, there are dozens of models, few of which can distinguish BPD severity [8–11]. In the present study, we developed an early prediction model to distinguish the severity of BPD based on a five-year cohort at a level IV NICU. To our knowledge, this is the first prediction model for BPD severity based on a large sample in a Chinese population. The developed model enables an early prediction of BPD severity at 28 days of oxygen treatment.

In the present ordinal logistic regression analysis, we found that GDM, initial FiO_2_ value, invasive ventilation, acidosis, hypochloremia, CRP level, PDA and positive respiratory culture were independent risk factors for BPD severity.

The present univariable and multivariable analyses suggest that maternal DM may aggravate the clinical severity of BPD. Meta-analysis showed that hyperglycemia during pregnancy may indicate adverse outcomes in preterm infants, such as preterm birth and respiratory distress syndrome [21]. However, in the previous literature, controversy exists regarding whether GDM is related to BPD. A meta-analysis based on studies conducted in developed countries found no difference in the incidence of BPD between infants with and without GDM, and contrasting results have been reported in China [14, 22]. Given that poor DM management may lead to hormonal disorders and unstable glucose levels in neonates, differences in results between these studies may be related to disparities in pregnancy management. As GDM was a significant risk factor for BPD severity in the present study and there is no relevant previous study on BPD severity, further attention should be given to how maternal diabetes influences the infant’s lung development.

Respiratory support plays an important role in BPD development. Non-invasive methods were recommended by randomized controlled trials and meta-analyses to reduce the risk of BPD [21, 23]. Consistent with these studies, we found that intubation in the DR was related to BPD severity, which is probably because of its ventilator-induced lung injury, volutrauma and barotrauma [24]. We also found an initial high FiO_2_ to be an independent risk factor. Hyperoxia can cause the release of reactive oxygen species, resulting in both cellular and molecular damage to the lung. Thus, attention is needed to avoid intubation in DR. Strategies, such as less-invasive surfactant administration and CPAP, have already been proven to improve respiratory outcomes [24]. In addition, a recent study showed that better lung recruitment maneuvers may reduce the rate of intubation in DR [25].

Acid-based and electrolyte disturbances have been previously identified as risk factors for the development of BPD [26]. Hypochloremia and hyponatremia were related to BPD severity in the present study, which is consistent with previous studies [27]. Several studies have linked electrolyte disturbances to the use of diuretics; however, in the present study, we collected data within the first 14 days of life, when diuretics are seldom used. Thus, electrolyte disturbances in this cohort had a unique pathogenesis, rather than being secondary to therapy. Electrolytes are of paramount importance to both intra- and extracellular fluid balance as well as the normal function of cells. Some researchers suggest that electrolytes disrupt the normal function of cells, while others suggest that they may elevate arginine vasopressin levels, leading to pulmonary edema and, eventually, BPD [26, 28]. Most of the patients with BPD experienced acidosis to some extent in this study. According to the previous literature, acidosis may reflect immature renal function, complex metabolism, or respiratory failure [29–31]. Although the mechanism remains unclear, the present study results suggest the importance of close follow-up and the correction of the pH value and serum electrolyte levels in preterm infants.

Inducing systemic inflammatory responses, sepsis has been shown to increase the BPD incidence rate. In the present study, LOS was related to BPD severity, which is consistent with previous studies [32]. However, in the multivariable analysis, the inflammation indicator CRP replaced LOS as an independent risk factor. This is consistent with findings by Yang et al., which found that early CRP levels may be used as an early diagnostic marker for mild-to-severe BPD with an AUC of 0.867 [13]. Although culture remains the gold standard for sepsis, the positive rate is low, especially in early life. With advantages of convenience and quickness, CRP has proven to be a good biomarker for neonatal sepsis. In recent years, new inflammation indicators, such as presepsin, have been proposed to be more promising biomarker of neonatal sepsis [33].

Pulmonary infection also plays an important role in BPD pathology. Both Gram-positive and Gram-negative pathogenic bacteria were associated with an increased risk of greater BPD severity in the present study, while Gram-negative bacteria were more related to the outcome. In the current decade, researchers have focused on the role of respiratory flora in BPD; previous studies have shown a strong association between Gram-negative bacteria and poor respiratory outcomes in preterm infants [34, 35]. Lipopolysaccharide, a major component of the outer membrane of Gram-negative bacteria, interacts with cell-surface receptors, leading to an increased secretion of inflammatory mediators, prompting an inflammatory cascade and causing diffuse lung tissue damage and alveolar simplification [36]. Thus, investigations on methods to reduce Gram-negative bacteria in the airway of chronically high-risk preterm infants are critical.

In summary, the role of inflammation in the pathogenesis of BPD has been firmly established, and both localized and systemic infections were revealed as independent risk factors in the present study. Thus, clinicians should pay more attention to optimal anti-infection treatments, as well as strict nosocomial infection control.

Despite the role of infection in BPD, antibiotics may be a double-edged sword in clinical management. Ting et al. found that among infants without culture-proven sepsis, more antibiotic exposure was associated with adverse neonatal outcomes [37]. Consistent with this, Fajardo et al. found that antibiotic treatment for more than five days in preterm infants with negative cultures was associated with increased BPD risk [38]. In the present study, we found similar results that prolonged antibiotics increased the risk for BPD severity. According to previous studies, this may be because antibiotic use in preterm infants may lead to disruption of the neonatal microbiome development and an increased risk of pulmonary development failure [39, 40]. However, a recent study using stepwise hierarchical analyses suggested the existence of factors confounding the association between early antibiotic exposure and BPD. In other words, antibiotic treatment in early life is more likely a sign of illness severity than a true contributor to BPD.

Factors impeding alveolar maturation, such as BW and GA, are believed to be of prime importance in the pathology of BPD. Contrary to general impressions, the present multivariable analysis indicated no significant differences in GA among BPD severity groups. Since we restricted the cohort to patients with BPD, this result might be partly due to sample homogeneity. Most patients in this cohort were in the saccular stage [41]. Thus, postnatal factors may have displaced GA as the main risk factor.

In previous studies, male preterm infants were more prone to developing BPD than female preterm infants. Estrogen may promote lung maturation and stimulate the expression of PS [42]. In the present study, male neonates accounted for 61% of BPD cases, verifying the susceptibility in boys; however, sex was not related to the clinical severity of BPD according to our data.

Most existing prediction models were developed using traditional statistical methods. However, with computer science widely utilized in clinics, many centers have also attempted to establish BPD prediction models based on artificial intelligence technology in recent years. In 2021, a center in Denmark proposed an artificial intelligence model based on support vector machine learning to predict the occurrence of BPD; by combining postnatal clinical characteristics and exhaled gas nitrogen content, the accuracy reached approximately 90% [8]. Another center established a machine learning model for predicting BPD and severe BPD using clinical data and genomics, with an AUC of 0.872 [9]. Our model performed as well as the previous ones, and we have some advantages. First, the previous study took metabolomic and genomic indicators into account in the models, and the huge cost might render it difficult to popularize the previous models. In the present study, we selected easily accessed clinical factors and validated the findings by ordinal regression analysis, increasing the interpretability of our models. Second, we used different machine learning modeling methods, further supporting the reliability and generalizability of our models. Moreover, our sample size is larger than those of past machine learning studies, which can avoid overfitting risk and thereby biased results.

This study developed an early prediction model using simple selected clinical and laboratory factors. First, all the factors involved are easy to access, which means that the model can be applied in most hospitals even with variability in medical quality. Second, this study also drew attention to some factors related to BPD severity in our own population, which can help improve our clinical management.

The present study has some limitations. First, as a retrospective study, we missed some factors that might be related to BPD, such as chorioamnionitis, early extrauterine growth retardation and early fluid management. We may add the above factors in future prospective studies. Second, as a single-center study, the model still needs external validation in a new group.

In conclusion, in this single-center study, GDM, initial FiO_2_ value, invasive ventilation, acidosis, hypochloremia, CRP level, PDA and positive respiratory culture were independent risk factors for BPD severity and we developed machine learning models with good performance. With simple factors involved, these models may become a useful tool in clinical practice for the early stratification of BPD, which can help clinicians choose individualized treatment protocols and follow-up strategies in the Chinese populations.

## Supplementary Information

Below is the link to the electronic supplementary material.

Supplementary file1 (DOCX 67 KB)

## Data Availability

The datasets generated during and/or analyzed during the current study are available from the corresponding author on reasonable request.
