# Supplementary material for: Risk factors and machine learning prediction models for bronchopulmonary dysplasia severity in the Chinese population
Source: World J Pediatr. 2022 Nov 10;19(6):568–76. doi: 10.1007/s12519-022-00635-0 (PMC10198877; doi:10.1007/s12519-022-00635-0)
Supplement: Supplementary file 1 — Supplementary file1 (DOCX 67 KB) [file 12519_2022_635_MOESM1_ESM.docx]

**Part 1 Data for very preterm patients without BPD.**

**Supple. Table 1 Characteristics comparison between patients with BPD and without BPD.**

|  | Non-BPD  n=1032 | BPD  N=471 | *P* |
| --- | --- | --- | --- |
| GA, w, mean (SD) | 29.7(1.8) | 28.3 (1.6) | ＜0.001 |
| BW, g, mean (SD) | 1390(329) | 1146(216) | ＜0.001 |
| Male, n (%) | 570(55.2) | 286(60.7) | 0.049 |
| SGA (< P10), n (%) | 40(3.9) | 15 (3.2) | 0.308 |
| Antenatal Corticosteroid Treatment, *n (%)* | 801(77.6) | 282(59.9) | ＜0.001 |
| GDM, *n (%)* | 112(10.9) | 73(15.5) | 0.014 |
| Maternal hypertensive disorder, *n (%)* | 348(33.7) | 148(31.4) | 0.408 |
| Maternal age (yr), mean (SD) | 31.0(5.3) | 31.6(4.6) | 0.019 |
| Delivery by Cesarean Section, *n (%)* | 494(47.9) | 237(50.3) | 0.404 |
| 1-min Apgar, median(P25-P75) | 8(7-9) | 7(5-8) | ＜0.001 |
| 5-min Apgar, median(P25-P75) | 9(8-10) | 8(7-9) | ＜0.001 |
| Intubation in the Delivery Room, *n (%)* | 257(24.9) | 139 (29.5) | 0.067 |
| Laboratory test: |  |  |  |
| WBC-max, ×103/μL, mean (SD) | 18.6±9.9 | 26.0±13.7 | 0.001 |
| CRP-max, g/L, mean (SD) | 11.6±15.5 | 26.8±34.6 | ＜0.001 |
| PH-min, mean (SD) | 7.30±0.07 | 7.21±0.08 | ＜0.001 |
| Na-min, mmol/L, mean (SD) | 132.1±4.0 | 129.9±3.8 | ＜0.001 |
| Cl-min, mmol/L, mean (SD) | 104.5±5.0 | 97.8±6.7 | ＜0.001 |
| Complications: |  |  |  |
| PDA, *n (%)* | 430(41.7) | 327(69.4) | ＜0.001 |
| NEC, *n (%)* | 112(10.9) | 48 (10.2) | 0.787 |
| Neonatal sepsis- |  |  |  |
| -Early onset, *n (%)* | 38(3.7) | 36 (7.6) | 0.002 |
| -Late onset, *n (%)* | 47(4.6) | 144 (30.6) | ＜0.001 |
| Respiratory Specimen Culture |  |  |  |
| Gram +, *n (%)* | 14(1.4) | 166(35.2) | ＜0.001 |
| Gram -, *n (%)* | 35(3.4) | 207(43.9) | ＜0.001 |
| Early Treatment: | |  |  |
| Caffeine, *n (%)* | 737(71.4) | 453 (96.2) | ＜0.001 |
| Postnatal Corticosteroid Treatment, *n (%)* | 10(1) | 70(14.9) | ＜0.001 |
| Antibiotics Exposure without culture, *n (%)* | 959(92.9) | 410(87.0) | ＜0.001 |
| Prolonged Antibiotics Exposure, *n (%)* | 492(47.7) | 239(50.7) | 0.441 |
| Respiratory Support: | |  |  |
| Initial FiO2 , %, mean (SD) | 24.77±7.7 | 31.72±14.3 | ＜0.001 |
| Initial CPAP, *n (%)* | 341(33) | 114 (24.2) | 0.001 |
| Invasive ventilation,  *n (%)* | 358(34.7) | 273(58.0) | ＜0.001 |

*GA*, gestational age; *BW*, birth weight; *SGA*, small-for-gestational-age; *w*, weeks; *d*, days; *SD*, standard deviation; *GDM*, gestational diabetes mellitus; *yr*, years; *WBC*, white blood cell counts; *max*, maximum; *min*, minimum; *CRP*, C-reactive protein; *PH*, pH value; *Na*, sodium concentration; *Cl*, chloride concentration; *PDA*, patent ductus arteriosus; *NEC*, necrotizing enterocolitis; *CPAP*, continuous positive airway pressure; *FiO_2_*, fraction of inspiration O_2_; *Gram +*, gram-positive bacteria; *Gram -,* gram-negative bacteria

**Part 2 The model development based on 2018 NICHD criteria.**

In this part, we diagnosed and classified BPD according to the 2018 NICHD suggested redefinition: A premature infant (<32 weeks’ gestational age) with BPD has persistent parenchymal lung disease, radiographic confirmation of parenchymal lung disease, and at 36 weeks PMA requires 1 of the following FiO2 ranges/oxygen levels/O2 concentrations for ≥3 consecutive days to maintain arterial oxygen saturation in the 90%–95% range, the classification were shown in the follow chart [1].

| Grades | Invasive IPPV | N-CPAP, NIPPV, or nasal cannula ≥ 3 L/min | Nasal cannula flow of 1–<3 L/min | Hood O2 | Nasal cannula flow of <1 L/min |
| --- | --- | --- | --- | --- | --- |
| I | / | 21 | 22-29 | 22-29 | 22-70 |
| II | 21 | 22-29 | ≥30 | ≥30 | >70 |
| III | >21 | ≥30 |  |  |  |

We analyzed the selected eight risk factors according to the 2018 BPD classification by ordinal logistics regression and the results were shown in Supple. Table 2. We designated I, II, and III BPD as 1, 2, and 3, respectively. We can tell from the table that gestational diabetes mellitus (GDM), invasive ventilation, C-reactive protein (CRP) level and patent ductus arteriosus (PDA) was independent risk factors for BPD severity based on 2018 NICHD criteria.

**Supple. Table 2 Results of odd ratio for BPD severity levels (2018).**

| Variable | *P* ^a^ | Odds Ratio ^a^ | 95%CI |
| --- | --- | --- | --- |
| **GDM** | **0.037** | **3.463** | **1.081,11.096** |
| Initial FiO2 | 0.714 | 1.006 | 0.973,1.040 |
| **Invasive ventilation** | **0.012** | **6.391** | **1.505,27.136** |
| PH-min | 0.243 | 0.033 | 0.001,1.029 |
| Cl^-^_min | 0.140 | 0.971 | 0.933,1.010 |
| **CRP_max** | **0.002** | **1.018** | **1.007,1.029** |
| Gram- in culture | 0.219 | 2.132 | 0.638,1.727 |
| **PDA** | **0.034** | **6.291** | **2.920,13.553** |

GDM, gestational diabetes mellitus; FiO2, fraction of inspiration O2; PH, pH value; min, minimum; Cl-, blood chloride concentrations; CRP, C-reactive protein; max, maximum; Gram-, gram-negative bacteria; PDA, patent ductus arteriosus; 95% CI, 95% confidence interval

Words in bold font indicating significance between the groups.

^a^ adjusted odds ratios and *P* value for gestational age, gender and birth weight.

All eight selected clinical factors were entered into the LR machine learning model. ROC curves were drawn for the test set. The ROC of the models is shown in Supple. Figure 1 and the model-evaluation indexes were shown in Supple. Table 3.

**Supple. Figure 1 ROC of logistic regression model for BPD severity(2018)**


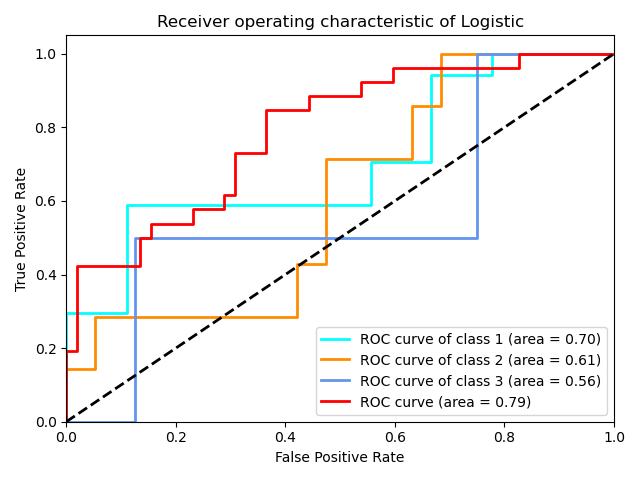


Fig.1 shows the performance for LR algorithm on test sets. Class 1 represents the mild BPD, Class 2 represents the moderate BPD and Class 3 represents severe.

LR, logistic regression; ROC, receiver operating curve; AUC, area under the receiver operating characteristic curve.

**Supple. Table 3 Model-evaluation indexes of logistic regression model for BPD severity (2018)**

|  | Precision | Recall | f1-score | AUC | 95%CI | SE |
| --- | --- | --- | --- | --- | --- | --- |
| I | 0.67 | 0.71 | 0.69 | 0.70 | (0.54, 0.86) | 0.083 |
| II | 0.29 | 0.29 | 0.29 | 0.61 | (0.46, 0.76) | 0.076 |
| III | 0.00 | 0.00 | 0.00 | 0.56 | (0.14, 0.99) | 0.217 |
| Total | 0.51 | 0.51 | 0.53 | 0.79 | (0.71, 0.87) | 0.039 |

**Part 3 Detailed data for the machine learning models**

Supple. Table 4 The detail indexes for all the machine learning models

|  |  | Precision | Recall | f1-score | AUC | 95%CI | SE |
| --- | --- | --- | --- | --- | --- | --- | --- |
| LR | Mild | 0.66 | 0.96 | 0.79 | 0.77 | (0.72, 0.81) | 0.024 |
|  | Moderate | 0.71 | 0.16 | 0.26 | 0.75 | (0.64, 0.86) | 0.057 |
|  | Severe | 0.50 | 0.40 | 0.44 | 0.83 | (0.72, 0.94) | 0.057 |
|  | Total | 0.67 | 0.66 | 0.59 | 0.86 | (0.79, 0.93) | 0.036 |
| GBDT | Mild | 0.77 | 0.76 | 0.77 | 0.71 | (0.61, 0.82) | 0.054 |
|  | Moderate | 0.39 | 0.54 | 0.46 | 0.65 | (0.49, 0.81) | 0.081 |
|  | Severe | 0.80 | 0.31 | 0.44 | 0.76 | (0.66, 0.86) | 0.052 |
|  | Total | 0.68 | 0.31 | 0.44 | 0.79 | (0.70, 0.88) | 0.045 |
| RF | Mild | 0.70 | 0.90 | 0.79 | 0.78 | (0.70, 0.86) | 0.042 |
|  | Moderate | 0.47 | 0.33 | 0.39 | 0.74 | (0.60, 0.88) | 0.071 |
|  | Severe | 0.75 | 0.23 | 0.35 | 0.72 | (0.49, 0.96) | 0.122 |
|  | Total | 0.65 | 0.66 | 0.63 | 0.84 | (0.77, 0.91) | 0.038 |
| XGB | Mild | 0.69 | 0.91 | 0.79 | 0.75 | (0.67, 0.83) | 0.040 |
|  | Moderate | 0.71 | 0.38 | 0.49 | 0.70 | (0.57, 0.83) | 0.066 |
|  | Severe | 0.50 | 0.20 | 0.29 | 0.76 | (0.66, 0.86) | 0.050 |
|  | Total | 0.69 | 0.69 | 0.66 | 0.85 | (0.77, 0.94) | 0.043 |

Reference:

1 Higgins RD, Jobe AH, Koso-Thomas M, Bancalari E, Viscardi RM, Hartert TV, et al. Bronchopulmonary Dysplasia: Executive Summary of a Workshop. J Pediatr. 2018;197:300-8.
